# Biological Obstacles for Identifying *In Vitro*-*In Vivo* Correlations of Orally Inhaled Formulations

**DOI:** 10.3390/pharmaceutics11070316

**Published:** 2019-07-05

**Authors:** Eleonore Fröhlich

**Affiliations:** 1 Center for Medical Research, Medical University of Graz, 8010 Graz, Austria; eleonore.froehlich@medunigraz.at; Tel.: +43-316-385-73011; 2 Research Center Pharmaceutical Engineering GmbH, 8010 Graz, Austria

**Keywords:** pharmaceutical aerosols, composition of epithelial lining fluid, lung deposition, pulmonary drug delivery, inhalers, patient compliance

## Abstract

Oral inhalation of drugs is the classic therapy of obstructive lung diseases. In contrast to the oral route, the link between *in vitro* and *in vivo* findings is less well defined and predictive models and parameters for *in vitro-in vivo* correlations are missing. Frequently used *in vitro* models and problems in obtaining *in vivo* values to establish such models and to identify the action of formulations in vivo are discussed. It may be concluded that major obstacles to link *in vitro* parameters on *in vivo* action include lack of treatment adherence and incorrect use of inhalers by patients, variation in inhaler performance, changes by humidity, uncertainties about lung deposition, and difficulties to measure drug levels in epithelial lining fluid and tissue. Physiologically more relevant *in vitro* models, improvement in inhaler performance, and better techniques for *in vivo* measurements may help to better understand importance and interactions between individual *in vitro* parameters in pulmonary delivery.

## 1. Introduction

Inhalation of aerosols has a long history in the treatment of lung diseases. It has the advantage that the drug is directly delivered at the level of the diseased cells. The lung is not easily accessible to medication because drugs have to be aerosolized and only particles in a certain size range can enter the respiratory tract. Inhalable particles should be in the micrometer size but different strategies have been developed in order to deliver also nanoparticles to the lung [1,2]. Inhaled therapies comprise medications for asthma (50%), chronic obstructive pulmonary disease (COPD, 15%), asthma/COPD (asthma-COPD overlap syndrome, ACOS, 8%), diabetes (9%), cystic fibrosis (4%), microbial infections (4%), and 5% narcotic analgesics [3]. Although in recent years inhalation was also advocated as promising non-invasive delivery route for systemic application (e.g., for small molecules and peptides), the main use of inhalation will remain local treatment. 

Depending on the disease delivery of drug compounds to a specific region of the lung is intended. Physical parameters that influence the delivery and biological action of aerosols include properties of the formulation (drug effects, onset of action, stability, side effects, drug interaction), inhaler device (airflow resistance, speed of aerosol plume, formulation characteristics, feedback/control mechanisms), and patient characterization (age, sex, race, device handling) [4]. Deposition in the lung, removal of particles by mucociliary clearance and phagocytosis by alveolar macrophages (AM), particle dissolution, and absorption by epithelial cells are relevant factors for transport of drugs to the target cells.

**Figure 1:**
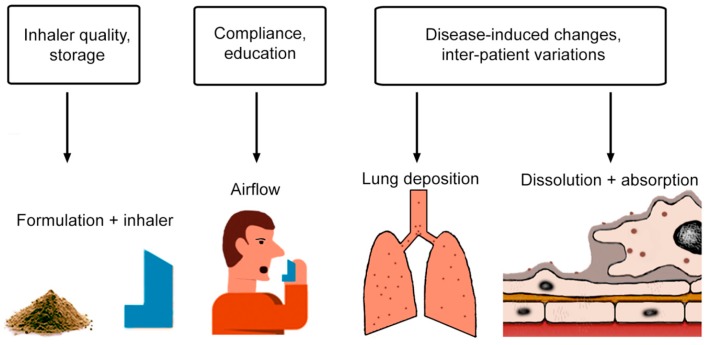
Overview of factors that influence relevant parameters for drug delivery to in the lung.

Changes in delivery may be caused by environmental conditions (humidity, temperature), quality of the inhaler, education and compliance of the patient, and disease- and patient-specific airway morphology, mucus viscosity, mucociliary clearance, phagocytic activity of AMs, and receptor expression (Figure 1).

For efficient development of orally inhaled products and good understanding of the role of formulation parameters it would be useful to be able to predict *in vivo* performance based on *in vitro* parameters in a similar form as for oral products, where methods for *in vitro*-*in vivo* correlation (IVIVC) are well established. IVIVC is given when *in vitro* dissolution and *in vivo* release of a dosage form are similar or have a scalable relationship to each other [5]. Standardized protocols have been established and, when certain limitations are respected, good prediction of the *in vivo* behavior of formulations or batches is possible for oral formulations [6]. Established *in vitro* methods for IVIVC for orally inhaled products, on the other hand, are missing. IVIVC is usually proven by comparison with plasma concentrations. Although this works well with oral formulations, there are numerous examples that *in vitro* tests were not predictive for outcome of pharmacokinetic (PK) studies for orally inhaled formulations [7]. *In vitro* parameters that have been suggested as predictive for *in vivo* performance are aerodynamic particle size, relevant for *in vivo* deposition, and *in vitro* dissolution and permeability for *in vivo* dissolution and absorption [8].

The review will discuss main parameters for variation of *in vivo* performance and mention *in vitro* methods currently used in development of orally inhaled formulations. 

## 2. Patient Inhaler Interaction

### 2.1. Role of Patient

Optimal interaction of patient and inhaler is crucial for drug delivery to the lung. This factor is also important for the interpretation of *in vivo* results.

Mainly three types of devices, pressured metered dose inhalers (pMDI), dry powder inhaler (DPI), and nebulizers are used to deliver the aerosol. pMDI contain drugs either as solutions or suspensions in a single propellant or propellant mixture. Patients inhale the aerosol in the plume produced by the device. Excipients such as ethanol or surfactants may be added to solubilize the drug or stabilize a drug suspension [9]. DPIs deliver powder and contain either pure drug particles or include particles blended to a carrier. Drug particles are inhaled by airflow generated by the patient. Nebulizers are jet or ultrasonic type and differ in the force used to generate the aerosol from the respective liquid. Depending on the model and the manufacturer, nebulizers generate 1–5 μm droplets. In contrast to pMDI and DPI, no effort by the patient to generate the aerosol is needed. Soft mist inhalers represent a new type of propellant-free inhaler that delivers a higher proportion of the emitted dose to the lung than pMDI or DPI. The device is easier to handle than pMDI because the mist from the inhaler continues for 1.5 s [10]. For further information on function, types, and use of inhalers the reader is referred to one of the many reviews focused on that topic (e.g., [11,12]). The majority of asthma patients use MDI, but the use of combinations of DPI and pMDI is also quite common (73% of patients) [13]. Use of nebulizer is focused on specific applications. 

Taking asthma as an example, non-adherence to therapy has been identified as the main factor for lack of disease control and increased mortality. The authors report that 65.2% fulfilled the criteria of suboptimal adherence, which was defined as <80% of prescriptions filled [14]. Even in clinical trials, 11% of patients fell into this category. Incorrect inhaler use of the inhaler by the patient also has dramatic effects., Rates of incorrect inhaler use were compared in studies between 1975 and 2014. The analysis showed that most common errors for pMDI were lack of hand-breath coordination (41–49%), errors in depth/speed of inhalation (40–47%), and lack of post-inhalation breath hold (42–47%) [15]. Users of DPI committed the following errors most often: incorrect preparation of the inhaler (26–33%), no full expiration (42–50%) and no post-inhalation breath hold (33–40%). In a systemic review of 38 studies reporting inhaler misuse by asthma and COPD patients >18y, it was stated that rates of incorrect use were mostly in the range of 0–20%. pMDI, DPI, and respimat inhalers were included in the review and there was no trend for higher rates of misuse in a given inhaler type [16]. Another study on inhaler misuse by adult asthma and COPD patients reported overall error rates of 50–100% with critical errors in 14–92%. Heterogeneity between the studies was high (>90%) [17]. The high number of different definitions for “critical errors” used in the studies may explain the different results [18]. Effects of inhaler mishandling on *in vitro* parameters have been observed in several studies. The exhalation into the Turbuhaler^®^ caused inconsistent fine particle fraction (FPF) of three subsequent shots [19]. Inappropriate use, lack of priming and incorrect inhalation procedure, may decrease the FPF by ~50%. Omission of shaking of pMDI before use reduced emitted dose (ED) and FPF of salbutamol by 25% and 36%, respectively, while storage of the pMDI upside down reduced FPF by 23% [20]. 

Disease-linked problems such as inability by patients to generate the airflow required for detachment of the drug particles from the carrier or aerosolization of the drug particles in DPI are expected to play a minor role because DPIs with required airflow between 37 and 111 L/min are available and a DPI suitable for the patients’ respiratory condition can be prescribed [21]. Differences in the breathing pattern between patients and healthy individuals, on the other hand, cannot be accounted for by the inhaler. It is known that breathing pattern is altered in patients in the way that the time to reach the maximal tidal lung volume and the time for exhalation are longer compared to a healthy person [22]. Changes in airflow did not affect all airways to the same extent. Maximal airway velocity was not affected in generation G5 of the bronchial tree (corresponding to the end of the upper airways with 3.5 mm diameter) but strongly affected in G6-1 to G7-3 (smaller airways with 2.2–2.7 mm diameter) at light and moderate exercise [23].

### 2.2. Role of Inhalers

Drug delivery is markedly influenced by type and properties of the inhaler and reproducibility of aerosol delivery is a critical parameter for device performance. Velocity of evaporation of solvent and co-solvents is important for aerosol delivery by pMDI. Delivery decreases with increasing ethanol concentrations because evaporation decreases and droplets remain larger, which results in increased oropharyngeal deposition [24]. The interplay between plume velocity and inhalation airflow is complex as turbulences can be generated that increase deposition in the lung [25]. For DPIs, aerosol deposition depends on ED, aerodynamic particle size distribution (APSD), and device resistance [8]. One problem in the comparability between inhalers is that the FPF is not a constant value but depends on airflow. High inspiratory flow rate (IFR) will increase oropharyngeal deposition by impactation of particles [26]. In particle delivery from Turbuhaler^®^ and Novolizer^®^ FPF increases with airflow while for Diskus^®^, Cyclohaler^®^ and Elpenhaler^®^ delivery is independent from airflow. As increase in delivery upon increased airflow is needed to compensate the shift of deposition towards larger airways, IFR dependent delivery is favorable from a theoretical point of view. IFR is usually linked to internal resistance of the inhaler. At high internal resistance the FPF increases with IFR. The high resistance decreases the maximally attainable flow rate thereby reducing oropharyngeal deposition. Ideal inhalers would have high and consistent IFR-dependent FPF. 

Uniformity of ED and APSD are parameters of the quality testing of inhalers according to the respective regulatory agencies like European Medical Agency (EMA), United Stated Pharmacopeia (USP) ([27,28]). Dosage uniformity is met if “not less than 9 of the 10 doses are between 75% and 125% of the label claim, and none is outside the range of 65% to 135% of the label claim” [29]. For pMDIs, a fixed airflow rate of 28.3 L/min has to be generated from the mouthpiece but there are no such fixed indications for DPIs. The sampled airflow rate should be selected such that it generates a differential pressure drop of 4.0 kPa across the inhaler, tested at duration to withdraw 4.0 L of air [30].

Different marketed pMDIs (Victory, Non-US Ventolin, Assal, Xeneric-S, Sacrusyt) with the same labelled dose of salbutamol delivered different doses and mean respiratory mass [31]. What is less expected is the fact that variation between batches of marketed inhalers can be considerable. Means of drug plasma levels in healthy volunteers after inhalation of aerosols from three different batches of Advair Diskus^®^ were not bioequivalent according to the existing definitions [32]. Variation of C_Max_ concentrations was 1.55 for fluticasone propionate and 1.62 for salbutamol, while AUC_0-∞_ values varied by a factor 1.29 and 1.35 for the two drugs. Older Diskus^®^ inhalers showed a 19.3–23.2% lower AUC of fluticasone than inhalers from a more recent production [33]. Dependency of ED from airflow is a typical parameter for evaluation of DPI. While in simulated patient use at 60 L/min differences in ED of Turbuhaler^®^ were not significant, delivered dose at lower airflow showed higher variations [34]. The importance of the variable ED is unclear as the majority of patients can generate airflow of 60L/min. Other studies, for example, one on Spiromax^®^, report only small variations of ED between batches under moisture exposure and after mishandling of the inhaler [35]. Also, variations of ED and particle size distribution in various pMDIs (Flixotide^®^, Pulmicort^®^, Alvesco^®^, Qvar^®^) showed small variations over the lifespan of the inhaler for budesonide, ciclosonide and beclomethasone. Variations of ED between 63–106% were only observed for one product (fluticasone 125) [36]. The authors suggested that the measurement technique of ASPD might explain differences between FPF values. Losses in FPF collected in the impactor were between 40–50% of label claim for beclomethasone, while with laser diffraction technique more than 70% were recovered. 

In addition to performance in standard conditions, stability tests according to the guidelines of the EMA consist of assessing these parameters under different conditions, e.g., 20 °C/60% relative humidity (RH) and 40 °C/75% RH [37]. MMAD, fine dose fraction (FDF) and emitted fraction (EF) should be indicated, but other parameters (physical, biological microbiological) should also be included. The guidelines also request information on effects of inappropriate handling of inhalers. For pMDIs the effect of storage of the inhaler in different positions needs to be tested with vertical upright, diagonal, horizontal and inverted upright orientation as common values. If secondary material is needed for DPIs, stability needs to be performed in the unwrapped product and after removal of the packaging. Due to the complex technology even for marketed products it may happen that certain batches of specific pMDI and DPI products (e.g., Bricanyl, Pulmicort, Ventolin, Asmol) are recalled from the pharmacies.

The different environmental conditions affected *in vitro* performance of inhalers and several studies reported prominent decrease on FPF delivered from DPI when stored for several weeks at 40 °C/75% RH. FPF of terbutaline from Turbuhaler^®^, fluticasone propionate/salmeterol from Accuhaler™, and Budesonide/formoterol from Easyhaler^®^ decreased between 40–60% [38,39]. In contrast to that, decreases in FPF under these conditions were around 10% for budesonide/formoterol in Turbuhaler^®^ and fluticasone/salmeterol in Spiromax^®^ [38]. Authors mentioned that increase in moisture appears to be more relevant for changes in FPF than the increase in temperature. It is hypothesized that the decrease in FDF by humidity is caused by increased adhesion of the drug particles to the carrier and higher interparticle forces due to increased capillary interactions. Furthermore, lactose monohydrate may dissolve and recrystallize under forming solid bridges with the drug particles [40]. Also, moisture-induced changes in *in vivo* effects were noted. Solutions and suspension formulations of inhaled corticoids (beclomethasone dipropionate or fluticasone propionate) behaved differently, but for all pMDIs, lung deposition decreased to 50% with increasing RH [25]. Higher temperature, on the other hand, increased lung deposition for solution pMPI because the drop-in temperature upon evaporation and absorption of water are less pronounced. This effect was not observed for pMDI containing suspensions. 

## 3. Particle Action in the Deep Lung

Particle sizes between 1–5 µm are indicated as optimal for deposition in the lung and the fraction of particles <5 µm is regarded as critical performance parameter for orally inhaled formulations. Recommendation of optimally respirable particles is based on deposition pattern calculated by International Commission on Radiological Protection (ICRP) model [41]. It is assumed that larger particles cannot reach the deep lung and that particles <1 µm are exhaled to ~100% due to their low settling velocity. Deposition of <0.01 µm particles is due to diffusive deposition and of 5–10 µm particles due to sedimentation and impactation [42]. Several other models exist that suggest similar size preferences for deposition (see available reviews for instance [43,44]). Experimental data for establishment of the models were generated with environmental particles, generally insoluble metal and metal oxide particles, such as iron oxide, and uranium particle with sizes of 0.1–7.5 µm and particle density of 2.6–3.2 g/cm^3^ [45]. Environmental particles differ in terms of solubility, change in moisture, and particle density from particles in therapeutic aerosols. Further, the model does not account for forced inhalation and breath hold. It may, therefore, be possible that data generated with this model are not predictive for inhalation treatment. It is, for instance, likely that particle agglomeration due to humidity occurs for pharmaceutical aerosols to greater extent than for insoluble particles [46]. 

### 3.1. Deposition

Deposition *in vivo* is difficult to quantify due to the three-dimensional nature of the bronchial tract and commonly used 2D technology. Gamma scintigraphy is the routinely used method to determine particle deposition. The amounts of radio-labelled material is low (1 MBq) and image acquisition time is short (3 min) [47]. Single photon emission computed tomography (SPECT) imaging needs radioactivity of 40 MBq and acquisition of images takes 15–30 min. Long imaging times are unwanted not only because of radioactive exposure but also because the radioactive label can detach from the drug and the signal, therefore, not correspond to the location of the drug. Positron emission tomography (PET) combines low exposure to radiation with 3D images and short image acquisition time. This technology, however, requires specific equipment to produce the radioactive aerosols (cyclotron) and is very expensive. Comparison between 2D (gamma scintigraphy) and 3D imaging (SPECT) showed, however, that differences between aerosols regarding total deposition were similar and that the 2D technique, therefore, is suitable to determine lung deposition [48]. Fluticasone-HFA particles with MMAD of 2.0 µm were deposited to 24% and 22% according to 2D and 3D images and deposition rates for beclomethasone-HFA with 0.7 µm particles were 55% and 53% [48]. All imaging techniques have in common that they do not provide quantifiable distinction between deposition in small and in large airways. Suitability of deposition values for IVIVC is suggested by the finding that for salmeterol/fluticasone by Seretide Diskus^®^ inhaler good correlation between FPF and lung deposition was seen [49]. Both parameters, FPF and lung deposition, decreased to ~50%, while there were no differences for formoterol/budesonide delivered by Turbuhaler^®^ in both parameters. 

The extent of deposition in mouth and throat is the main reason for inter-individual differences [50]. Furthermore, the left lung may have higher deposition despite the fact that the right lung receives more particles [51]. The higher deposition is due to the smaller cross diameter and the more curved airway structure. The number of airway generations to reach the alveoli is not the same throughout the lung [52]. Changes by diseases were reported in the way that the number of small airways is significantly different between COPD and healthy individuals [53]. Alterations in asthma involve large and small airways, while in COPD the small airways are most affected [53]. In addition to bronchoconstriction, bronchial dilation has also been reported. In cystic fibrosis, dilated bronchi and obstruction by mucus plugs are prominent [54]. The pathologic changes start in the small airways but affect the entire bronchial tree at later stages. Abnormal airway branching, however, was also described in smoking subjects, patients with heart disease, lung cancer, stroke, and other diseases [54]. It has also been shown that the average dose/surface area of ≥0.01 µm particles at the bifurcation of the trachea is 100–500 times higher than at other regions of the lung [42]. It is hypothesized that the preferential localization of bronchial carcinoma in these areas is due to the fact that particles containing environmental toxicants also deposit there [55]. Inhalation studies with radio-labelled ^99^Tc-sulfur colloid particles (0.22 µm integrated in 5.4. µm particles) further showed that particle deposition is not homogenous in the lung and altered in pulmonary diseases [56]. Compared to healthy volunteers, the number of regions not showing the radioactive signal (cold spots) was increased in cystic fibrosis patients, while regions with high accumulation of radioactivity (hot spots) were not significantly changed. Further, some reports mentioned that aerosols deposit to greater extent in healthy than in diseased areas of patients with lung diseases [57]. The uneven distribution poses another problem for correlation between particle properties and biological effects. 

Deposition *in vivo* is the result of landing of the particle on the epithelium and clearance. Mucociliary clearance describes the transport of particles in mucus from the lung to the oral cavity [58]. Particle transport is 100–300 µm/s in humans and cilia of the respiratory epithelium are the driving force for the movement. Cilia are surrounded by fluid with low viscosity (periciliary layer, PCL) and, when extended, have contact with the more viscous overlaying mucus layer. With the forward stroke the cilia move the mucus layer, while with the recovery stroke, where cilia are bent and not in contact with the mucus layer, no transport of the mucus occurs. Surface and amount of mucus presented in the smaller airways are much higher than in larger airways. In order to prevent overload with mucus length and density of cilia are higher in the larger airways. In addition to the movement of the cilia, which can be impaired by genetic mutations, infections, and action of environmental pollutants, mucus viscosity represents the main factor for decreased mucociliary clearance [59]. Elasticity and viscosity are interrelated and decrease of mucus viscosity beyond the optimal value by application of mucolytic agents does not increase mucociliary transport further but results in decrease. The optimal difference between periciliary fluid and the mucus is a 10× higher viscosity of the mucus [60]. Further information on the interaction of elasticity and viscosity of mucus is available elsewhere (e.g., [61]). Impairment of mucociliary clearance in obstructive lung diseases is supposed to be caused by hypersecretion of mucus and increased mucus viscosity [59]. These changes are prominent in all cystic fibrosis patients, while alterations in COPD patients were seen in smokers, not in ex-smokers [62]. Measurement of the mucociliary clearance in humans uses ^99m^Tc colloids and can only performed in few centers dedicated to this technology. Coughing is a mechanism to increase mucociliary clearance and has diagnostic value (first manifestation and prediction of future exacerbations) in COPD [63].

### 3.2. Clearance

Phagocytosis by AMs represents the main mechanism for clearance in the alveoli [64]. Only ~3% of the alveolar surface is covered by AMs but due to their movement with a speed of 2 ± 1.5 µm/min they can patrol the entire alveole within one day. This estimation corresponds to the *in vivo* finding that phagocytosis of 3–6 µm non-biodegradable particles is achieved within 24 h. The extent of removal is linked to the number of particles deposited and dependent on motility and number of AMs. External stressors, smoke, and environmental pollutant, as well as diseases and inflammation, inhibit normal macrophage function. COPD and asthma significantly reduced phagocytosis of various bioparticles (*Haemophilus influence*, *Staphylococcus aureus*, yeast zymosan bioparticles), and of apoptotic cells (termed efferocytosis) by AMs [65,66]. In contrast to the murine cells, uptake of latex particles and of *Staphylococcus pneumoniae* was not impaired after smoke exposure in human AMs [67]. 

Despite pronounced morphological (airway structure) and physiological (clearance, breathing pattern) differences, particle deposition based on imaging in healthy individuals and patients was reported similar. Formoterol-HFA from pMDI was deposited to 31% in healthy, 34% in asthma, and 35% in COPD patients [68]. The corresponding values for beclomethasone dipropionate/formoterol fixed dose combination were 34% in healthy, 31% in asthma, and 33% in COPD patients, and 55% in healthy, 56% in asthma, and 55% in COPD patients for beclomethasone dipropionate/formoterol fumarate as DPI formulation [69]. Non-pharmaceutical 0.5–1.5 µm ^99m^Tc particles were even deposited to higher extent in asthma patients. Longer residence time due to slower breathing and constriction of airways was proposed as a major reason for that [70]. Combination of the various disease-induced influences (deposition, clearance, and breathing pattern) complicates the prediction of changes in the deposition pattern of particles compared to healthy subjects.

According to the ICRP model, particles <1 µm do not deposit efficiently and a considerable fraction of extrafine particles (<2 µm) should be exhaled. It has, however, been reported that deposition of 0.7 µm beclomethasone-HFA was 55% [48], which contradicts the model. Hygroscopic growth of formoterol fumarate and beclomethasone dipropionate particles was 1.4 and 1.7 for the p10 fraction [71]. This suggests that hygroscopic growth caused deposition of the smaller particles. The different extent of hygroscopic growth complicates predictions on deposition based on data from dry formulations. An analysis of 18 studies including data from 32 inhaled formulations analyzed by gamma scintigraphy in healthy individuals and patients reported that the ratio of exhaled to deposited particles was independent from MMAD. Furthermore, deposition increased with decreased MMAD in a similar deposition pattern for healthy and diseased lungs [72]. Moisture-induced particle growth may also explain why breath hold did not increase lung retention as observed for environmental particles. ^99m^Tc containing NaCl aerosol (MMAD 0.55 µm) was exposed to humidity of 95% and inhaled by volunteers, with and without breath holding. Total lung deposition was 47 ± 4% with breath hold and 27 ± 4% without [73]. The high hygroscopy of NaCl lead to an increase in the size of a factor of two and increased lung deposition, as expected. On the other hand, however, fast clearance was higher with breath hold than without. This effect is most likely due to the fact that mucociliary clearance is the main clearance mechanism in the upper airways and particle removal by AMs in the alveoli. 

Moisture-induced effects have been determined by comparison of size distribution of inhaled and exhaled aerosols and deposition *in vivo* using NaCl particles. Absorption of moisture and increase in particle size due to hygroscopy occurs in the frame of milliseconds (0.1 sec for particles <1 µm) to seconds (5–10 s for 5 µm NaCl particles). Deposition was linked to hydrophilicity of the particles. While hydrophilic NaCl particles were maximally deposited at 1 µm size, maximal deposition of the more hydrophobic terbutaline particles was 5 µm [74]. It was concluded that hygroscopic growth plays a role in the deposition of particles < 0.5 µm Mass Median Aerodynamic Diameter (MMAD) can be exploited to increase deposition of submicron- and nanosized particles [75].

### 3.3. Drug Absorption

Particles after deposition are in contact with the epithelial lining fluid (ELF), the exact composition of which is unknown. For wetting and solubility of poorly water-soluble drugs, the concentration of lipids is expected to play a major role. The main lipids in ELF are phospholipids such as dipalmitoylphosphatidylcholine (DPPC). Neither bronchoalveolar lavage (BAL) nor induced sputum appears to be optimal to determine their concentrations in ELF. Induced sputum is performed only in specialized centers and successful only in 30–40% of the cases with particularly low success rates in healthy individuals that produce little sputum [76]. DPPC levels measured in induced sputum were 49.3 ± 20.1 µg/mL in healthy children. As DPPC represents ~50% of total phospholipids, the concentration of phospholipids would be 0.1 mg/mL [76]. BAL has repeatedly been used for qualitative and quantitative measurement of protein and lipid levels of the ELF. Calculation is based on the diffusion of urea from blood and values depend on volume of the instilled fluid and dwell time. The area that has been lavaged and the recovery rate of the lavage fluid also influence the results. The methodology of BAL has been improved over the years through the use of smaller volumes of the lavage fluid and shorter dwell times. This has increased the amount of ELF in the recovered BAL, which is nevertheless low (~10% of the total volume). As larger volumes and longer dwelling times were used in earlier studies the reported phospholipid contents were very low with 0.6–11.7 µg/mL [77], and only one older study mentioned a concentration of 84.1 ± 5.8 µg/mL [78]. In later studies higher total phospholipid content of 0.4 mg/mL was reported [79]. Alveolar wash of alveolar epithelium indicated total phospholipid concentrations of 30 mg/mL in rats [80] and 120 mg/mL phospholipid concentration in rabbits [81]. These data are in a similar range as the surfactant content of human lungs at birth of 100 mg/kg [82]. Surfactant content of newborns is 5–10 times higher than that of adults reducing the values determined in newborns to 10–20 mg/kg in adults. Under the assumption of a volume of ELF of 20–40 mL and a body weight of 70 kg in the standard adult, phospholipid concentrations may be estimated between 1.75 and 7 mg/mL. Other authors indicate an extracellular surfactant pool of 10–15 mg/kg in adult mammals, which would correspond to 0.7–1g in a standard person and a concentration between 17 and 50 mg/mL in the ELF [83]. In summary, indication of phospholipid concentrations in the literature varied with a factor of 100 (0.6–50 mg/mL). In contrast to the highly variable data on lipid content, protein content is reported between 4.76 mg/mL and 9 mg/mL [64,84]. This is surprising because the same methodology has been used for calculation of these values.

Little information on disease-induced changes in ELF composition is available in the literature. Smokers had increased ELF volume and decreased albumin levels, while ELF of patients with interstitial lung disease had increased albumin levels according to BAL measurements [85]. Similarly, drug levels, with the exception of antibiotics (vancomycin, tobramycin, solithromycin, lascufloxacin, cefpodoxime) after oral application in human ELF are mainly unknown [86,87,88,89,90]. 

Epithelial cells, bronchial epithelial cells, and alveolar epithelial cells regulate drug absorption and access to target cells in lung tissue, e.g., smooth muscle cells. Most relevant pulmonary disease- induced changes are increased expression of mRNA and variation of β2-adrenergic receptor [91,92]. 

## 4. *In Vitro* Models to Assess Biological Processes

### 4.1. Deposition

The most commonly used *in vitro* techniques to predict deposition are impactor-based. The 5-stage Marple-Miller impactor (MMI) at 60 L/min, the 8-stage Anderson Cascade Impactor (ACI) at 60 L/min, the 4-stage Multi-Stage Liquid Impinger (MSLI) at 60 L/min, and the 7-stage Next Generation Impactor (NGI) with pre-separator at 60 L/min and 100 L/min are accepted by the Food and Drug Administration (FDA) to determine the aerodynamic particle size [93]. Due to insufficient size resolution MMI and MSLI are used less often. Holding chamber, spacer, and mouthpiece can be attached at the induction port of the impactors. By connection of the cascade impactors, that operate at constant airflow rates, to a breath simulator physiologically more relevant conditions can be generated [94]. Alternatives to the impactor-based measurements include instruments that measure time of flight like Single Particle Aerosol Mass Spectrometry (SPAMS) and Laser diffraction techniques. For critical comparison of the different techniques the reader is referred to review articles dedicated to this topic, for example [95].

Lung deposition can be experimentally determined using casts, which were initially made from cadavers but now are fabricated based on computed tomography (CT) or magnetic resonance imaging (MRI) images from live volunteers [96]. By combining images and casts models down to the 17^th^ generation of the bronchial tree (start of the respiratory zone) are possible.

Computational fluid dynamics (CFD) is a mathematical method to predict airflow pattern, track particles, and predict particle deposition. Simulation of airflow dynamics (inspiration/expiration), changes of airflow with age, and optimization of drug application are typical applications. Based on CT images, asthma related changes in deposition of 2.5, 5, and 10 µm particles were calculated. The 2.5 µm particles were deposited to higher extent in the upper lobes of patients with severe asthma compared to healthy lungs. A great advantage of CFD in combination with quantitative CT is the possibility to quantify functional changes induced by the specific morphological changes [97]. Deposition calculated for Budelin Novolizer^®^ and fenoterol respimat soft mist inhaler showed good agreement with *in vivo* deposition [98]. Variation of anatomy and airflow enabled patient-specific simulation of drug delivery for a specific inhaler [99]. The main disadvantage is that the developed model, due to dynamic forces on drug detachment and influence of jet/turbulence velocity at the mouthpiece, is inhaler-specific. 

### 4.2. Clearance 

Mucociliary clearance can be determined by measuring movement of fluorescent beads on the surface of bovine trachea. Non-mammalian models (frog palate assay) can also be employed [61]. These conventional models lack directionality, but the more recently developed dynamic cultures, like the human small airway on a chip, overcome this limitation [100].

Phagocyte clearance is assessed by measurement of phagocytosis, using latex beads, sheep erythrocytes, or fluorescent pathogens (bacteria or yeast zymosan) with spectrofluorometry, confocal microscopy, flow cytometry, or imaging flow cytometry as readout [101].

### 4.3. Particle Dissolution

Dissolution in simulated gastric and intestinal fluids in combination with permeability across Caco-2 monolayers provides a good prediction of bioavailability for oral formulations. Simulated lung fluids may also be a tool to assess orally inhaled formulations and various fluids with similar pH and osmolarity but different amounts of antioxidant, lipid, and protein have been used [102]. Speed of dissolution has an importance on availability for poorly water-soluble drugs because not dissolved particles can be removed by mucociliary clearance and uptake by AMs. A variety of fluids and dissolution set ups have been published with buffered salt solution and addition of 0.02% DPPC as lipid compound used most often [102]. Problems for evaluation of the different fluids are not only lack of *in vivo* drug levels for validation but also the highly variable absolute concentrations reported in the literature for lipids in ELF. 

Even if DPPC can mimic effects of phospholipids, it cannot reproduce the different morphologies of pulmonary surfactant as lipid bi- or multilayers, tubular myelin, and uni- und multilamellar vesicles. BAL contains all these presentations of surfactant [103]. Curosurf^®^ the surfactant replacement produced from minced porcine lungs contains also lamellar body-like structures but lacks tubular myelin [104]. Interaction of drugs with lipid structures as representatives for plasma membranes have been extensively used and pulmonary surfactant as drug carrier has been suggested [105,106]. It may, therefore, be assumed that morphology of lipids also influences drug dissolution.

Particles used for the dissolution experiments were mainly collected from cascade impactors and dissolution in apparatus of the European Pharmacopoeia performed. Other collection methods and exposure set-ups have also been reported and been reviewed for instance by Floroiu et al. [107].

### 4.4. Drug Absorption

The most common model for assessment of permeability are Calu-3 bronchial epithelial cells and interaction of drugs on the cellular levels can be identified this way [108]. The membrane-based monocultures can be expanded by co-culture with other cell types of the lung and physiologically more relevant culture conditions, such as culture at an air-liquid interface and inclusion of mechanical stimulation [109]. Specific 3D models, preferentially used in lung cancer research, are spheroids, which can be composed of different cell types (organoids) or of a single cell type. Combination with microfluids by cyclic strain or fluid stress enhances differentiation of cells in 2D and 3D culture, and the low volumes used in microfluids represent better the interstitial fluids between cells in intact tissues.

More complex models, such as isolated perfused lungs and precision cut lung slices, are better representations of the *in vivo* situation but are more difficult to handle and present donor-specific differences [50]. The most commonly used explant culture, precision cut lung slices, are embedded in collagen gels or cultivated on semiporous membranes. 

Engineered tissues (reconstructed lung tissues) are composed of several cell types immersed in collagen or cultured on scaffolds [110]. Often mechanical or biochemical stimuli are added to increase the similarity to tissue *in vivo*. The first engineered lung tissues were built from decellularized lung matrix as tissue-specific scaffold. Newer techniques use bioprinting, where cells and bioactive cross-linkable materials are combined [111]. Hydrogels are composed of synthetic (e.g., polyethylene glycol, pluronic) or natural (collagen, chitosan, fibrin, gelatin, matrigel, alginate) polymers. The main challenge is that the cross-linkable polymer should not cause cell damage.

## 5. *In Vivo* Parameters for Drug Action

Plasma drug levels are the routine parameter for IVIVC and bioequivalence studies and are, therefore, also the most often used parameters for IVIVC of pulmonary applications.

### 5.1. Plasma Levels

Plasma levels can serve as substitutes for tissue concentration of drugs lacking metabolization in the lungs and characterized by immediate absorption. In this way budesonide and fluticasone propionate plasma levels can give a good indication on tissue levels [112]. Also, for salbutamol sulfate deposition by gamma scintigraphy and plasma levels showed very similar values (26.2% vs. 26.4%) [113]. Further, it has also been shown that deposition of budesonide is linked to local action. The direct link between deposition and *in vivo* action, on the other hand, is weakened by the fact that delivery of budesonide by DPI was twice that of pMDI but systemic availability was only 50% higher [114]. 

There are, on the other hand, several drugs, where blood levels and tissue levels are not expected to correspond. Basic molecules, for instance olodaterol, are subjected to intracellular retention by trapping in lysosomes. Long-acting muscarinic receptor antagonists (tiotropium, ipratropium bromide) and β2-receptor agonists (olodaterol), which have slow receptor off kinetics, and 21-OH corticosteroids that are esterified in lung tissues also fall into this category [115]. β2-receptor agonist tissue levels will also differ from plasma levels because they interact with lipid bilayers either by classic mechanism (salbutamol) through approach of the ligand from the aqueous phase, or by microkinetic interaction (salmeterol and formoterol) [116]. For formoterol, the plasma membrane merely acts as a reservoir and the drug diffuses out of the membrane and binds to the receptor, while salmeterol molecules approach the receptor from within the membrane. Higher ELF than plasma levels have been reported for antibiotics applied by the oral route. Levels of azithromycin, ciprofloxacin, clarithromycein, lascufloxacin, and solithromycin in ELF were 2.2, 2.5, 8.6, 10, and 29.6 times higher than in plasma [88,90,117]. This finding appears to be mainly due to accumulation of drug in AMs. Concentrations in ELF were much lower when corrected by the number of lysed cells or when determined by bronchoscopic microsampling instead of BAL [118]. AMs contained >10 times higher amounts of lipophilic drugs, like for instance ciprofloxacin, lomefloxacin, and solithromycin, than ELF. During preparation of the BAL, a variable amount of cells dies and the intracellular drug content is released. According to a study in rats, the differences between ELF and plasma levels will be even more prominent when antibiotics were applied by inhalation. In that study, small differences between EFL and plasma concentration of tobramycin were reported after iv application but levels in ELF were >200 times higher than plasma concentrations when the drug was applied as nebulized aerosol [119]. Cellular accumulation may also explain the 10 times higher tissue than plasma levels of inhaled fluticasone [120]. Disease-induced changes of drug dissolution in ELF may be expected for charged drugs. In general, the pH of ELF is acidic compared to plasma and pH of 6.8–7.1 were measured in preclinical models [121]. In human breath condensated exhalates, tracheal aspirates, and BAL pH values were 7.65 in healthy subjects and pH 5.23 in asthma patients (variation 4.5–8.5). Tracheal aspirates from neonates had pH 7.8. Taken together, lung epithelium appears to be exposed to considerable variation in pH. *Ex vivo* data from murine trachea showed that differences were linked to pCO_2_ [122]. Upon induced hyperventilation pH of ELF was ~7.5 and upon hypoventilation ~6.0. Solubility of positively charged β2-agonists and muscarinic antagonists may be affected by the variations in pH.

It is further likely that plasma levels do not reflect local tissue levels because of receptor binding. Membrane receptors for β2-adrenergic agonists are found on a variety of cells and cause relaxation of smooth muscles, production of surfactant by alveolar type 2 cells, decrease of mast cell degranulation and increase of mucociliary clearance [123]. Density of β2-adrenergic receptors in smooth muscle cells does not change along the different parts of the airways. M3 muscarinic receptors are expressed on smooth muscle cells, inflammatory cells, respiratory epithelial cells, and submucosal gland cells [124]. Therapeutic action of muscarinic antagonists includes muscle relaxation and decrease of mucus secretion, while anti-inflammatory effects have not been proven so far. Density of M3 muscarinic receptors is highest in smooth muscle cells of the large airways and decreases to small airways [123]. Inhaled corticosteroids (ICS) act via binding to glucocorticoid receptors, located in the nucleus, which are present in α and β form. Binding to the α-form has anti-inflammatory effects, while the β form, which is expressed in much lower levels, modulates the action of glucocorticoids [125]. The receptors are higher expressed in epithelial and endothelial cells with no prominent differences in density between large and small airways. In addition to their prominent anti-inflammatory effects, activation of glucocorticoid receptors increases density of β2-adrenergic receptors and decreases mucus secretion. The link between deposition of particles and effect is complex. In theory, a lower deposition in the small airway region should decrease the action of β2-agonists and ICS because receptor density is constant in the different regions of the bronchial tree. Lower deposition of M3 antagonists, on the other hand, should not affect the therapeutic effect because these receptors have higher density in the large airways. However, since the contribution of small airways to airway resistance is higher than that of large airways, the fraction of the drug deposited in the small airways is more relevant for the therapeutic effect [126]. 

Due to the great relevance of the small airways for obstruction, bronchodilators should mainly be delivered at this part of the respiratory tract. Inflammation in COPD and asthma, on the other hand, affects the entire lung and ICS should, therefore, be delivered to all parts of the bronchial tree. The different target regions were the reason for the assumption that fixed dose combination products have suboptimal efficacy [127]. A study showing that fixed dose combinations for prevention of COPD exacerbations were more successful when they contained drugs with similar target region like dual long-acting muscarinic antagonists (LAMA)/ long-acting β2-agonists (LABA) combination than when they targeted different regions like ICS/LABA combination supported this hypothesis [128]. In general, however, treatment with fixed dose combination demonstrated equal efficacy to free combination of drugs in the clinical practice (e.g., [129]). Despite the great importance for delivery to the small airways, decrease in FPF with expected lower delivery to this region did not decrease the bronchodilatory effect. Salbutamol particles of 1.5 µm showed the highest deposition but 6 µm particles had the best clinical response. The higher amount of drug delivered by larger than by the smaller particles and the presence of uncoupled (not functional) β2 receptors in specific parts of the respiratory tract may explain this finding [130]. Uncoupled β2 receptors have been demonstrated in cells isolated from asthma patients but the relevance *in vivo* is still debated [131]. It may be assumed that extent of deposition is not always linked to therapeutic effects because several factors interact. 

Regarding the relevance of plasma levels it can be further stated that they do not reflect deposition because absorption from the large airways contributes only a little to plasma concentrations because the surface area (2–4 m^2^) of the tracheobronchial part is much smaller than that of the alveoli (140 m^2^; [132]). Therefore, drug plasma levels cannot be advocated as representative for drug concentrations in tissue and differences between therapeutic efficacy and plasma concentrations may occur.

### 5.2. Therapeutic Efficacy

Plasma concentrations are the common parameters in IVIVC but not usually determined in clinical trials. Typical readout parameters for clinical efficacy include improvement of pulmonary function (forced vital capacity (FVC), forced expiratory volume in 1 s (FEV1), and time to first severe exacerbation). Reduction of asthma exacerbations as parameter for patient adherence to inhaled medicines showed a clear correlation of poor adherence and increase of exacerbations. However, reduction of exacerbations was not linearly correlated with use of inhaled corticosteroids and 75% adherence was sufficient to reduce exacerbations to the same extent as 100% adherence. Furthermore, intermittent dosing of ICS was reported to have similar beneficial effects to continuous dosing [133]. Correlation between *in vivo* effects and moisture-induced changes in formulations has not been reported and there were no effects of ambient humidity on any clinical outcome of DPI therapy [134]. Asthma control using pMDI with salbutamol and DPI with terbutaline in hot humid regions was equally well [135]. Lack of effect of decreased FPF on therapeutic effect may be due to the fact that differences in deposition were not pronounced enough. Alternatively, receptor properties may play a role. In addition to receptor distribution, the response curve of receptors may explain this effect. The most commonly used anti-asthma drugs, ICS, muscarinic antagonists, and β2- agonists, have a sigmoidal response curve and higher doses will result in greater effects only if the lower dose caused an effect. Furthermore, when the plateau is reached, no further increase of the response will be observed [19]. For β2-agonists a twofold increase of the dose results in increased bronchodilating effect, while for ICS a fourfold increase in the dose is needed to cause a significantly different effect. Lack of sensitivity to the receptor subtype may be an additional factor for variable action of β2-agonists [136].

## 6. Conclusions and Outlook

It can be concluded that a potential link between *in vitro* parameters and *in vivo* performance for orally inhaled drugs is difficult to assess due to poor patient adherence to therapy and frequent errors in inhaler use. Variations in the delivery from inhalers and effects of inhaler mishandling by the patient, disease-related changes in airway morphology, pH and viscosity of airway lining fluid and receptor distribution will also induce some variation. The variations render identification of specific parameters important for IVIVC difficult. Using plasma levels as parameter for IVIVC and substitute for lung tissue concentrations cannot be recommended for all compounds. It has, on the other hand, to be considered that this parameter, compared to drug concentration in ELF, can relatively easy be determined. 

Several developments may improve the current situation in the future, while for others dramatic changes are not expected (Table 1). Incorrect inhaler use has been identified as problem in the treatment many years ago and better and more frequent training for caregivers and patients has been suggested. As the rate of incorrect inhaler use did not change markedly over the years, prominent changes in this parameter are not expected in the future. Fixed dose combinations should make treatment more patient-friendly and increase treatment adherence. Actually, one study reported that use of fixed drug combination inhalers improved adherence by 25% [137]. Improvement in inhaler quality, on the other hand, could lower the rates of incorrect inhaler use by patients. Physiologically more relevant *in vitro* models e.g., measuring deposition with impactors connected to mouth-throat models and breath simulation or CFD may serve for better evaluation of deposition. Co-culture systems may provide more insight into the interactions of cells at the absorption site. According to several studies AMs contain 10–500 times higher levels than plasma [90,117,138]. Potentially novel technologies for better determination of *in vivo* parameters (e.g., imaging, sensors) will be available. Endoscopic sensors have been developed for measurement of pH of ELF and are tested in animal *ex vivo* models [139]. Despite the fact that plasma levels are not identical to concentrations at the target cells, these data will remain the main reference for IVIVC. 

## Figures and Tables

**Table 1:** Expected changes in current problems with *in vitro-in vivo* correlation (IVIVC) in pulmonary drug delivery.

Parameter	Changes	Comments
Lack of patients’ adherence to therapy, incorrect use of inhalers	Improved information to patients about the need for treatment adherence and repeated training of patients	The problem is known for years and no improvement has been noted
Inhaler quality	Improved product quality, more detailed testing, fixed dose combinations	Improvement appears realistic
*In vitro* testing for lung deposition	Physiologically more relevant testing using impactors with breath simulation and mouth-throat model Testing under relevant moisture conditions Computation fluid dynamics simulations based on images	Both methods appear suitable to improve the prediction of deposition and to personalize the profile
Particle clearance and permeation *in vitro*	Cells and engineered tissues	Physiologically more relevant cellular models are being developed and improvement is expected
Dissolution	Identification of physiologically more relevant fluids	Studies are underway to solve these issues
*In vivo* parameters	Better imaging techniques for deposition New technologies for drug levels in ELF (sensors)	Improvement appears realistic
